# A Sensitive Approach to Managing Hypersensitivity Pneumonitis

**DOI:** 10.7759/cureus.29738

**Published:** 2022-09-29

**Authors:** Prashant Ahlawat, Prateek Upadhyay, Prateek Madaan

**Affiliations:** 1 General Medicine, Government Medical College & Hospital, Chandigarh, IND; 2 Anaesthesiology, Government Medical College & Hospital, Chandigarh, IND

**Keywords:** interstitial pneumonitis, pneumonitis, anchoring bias, extrinsic allergic alveolitis, interstitial lung disease, hypersensitivity pneumonitis

## Abstract

Hypersensitivity pneumonitis (HP), also known as extrinsic allergic alveolitis, is an immunologically mediated disorder that typically presents as a case of interstitial lung disease (ILD) in response to any identified or unidentified antigen. We present a case of a 46-year-old female with HP, who presented with fever and shortness of breath. Although negative by real-time reverse transcription-polymerase chain reaction (RT-PCR), her condition was initially diagnosed as COVID-19 clinically as a result of anchoring bias due to similar symptoms and radiologic features presenting in the pandemic. A detailed further probing into history revealed the diagnosis of HP due to cat hair, and hence, was managed accordingly.

## Introduction

Hypersensitivity pneumonitis (HP), also known as extrinsic allergic alveolitis is an immunologically mediated disorder in response to any identified or unidentified antigen that typically presents as a case of interstitial lung disease (ILD) [1]. It is characterized by a delayed type of allergic reaction due to repetitive or long-term exposure to a sensitized physical agent, biological antigen such as organic dust (of animal or plant origin), or seldom, chemical agents [2]. Although previously classified into acute, subacute, and chronic types, a recently proposed classification by Lacasse et al. categorizes HP into two subtypes, namely acute HP and chronic HP [3]. In acute HP, the symptoms may appear a few hours after exposure to the sensitized antigen and are usually flu-like such as shortness of breath, cough, fever with chills, etc.; whereas chronic HP is more insidious in onset with symptoms of worsening dyspnea, fatigue, dry cough, loss of appetite, etc. [4,5]. Additionally, the 2020 guidelines from the American Thoracic Society, Japanese Respiratory Society, and Asociación Latinoamericana de Tórax (ATS/JRS/ALAT) categorize subtypes of HP as nonfibrotic or fibrotic, as these subtypes are easier to distinguish and correlate better with clinical outcome [6].

Eliciting a thorough patient history, with a particular interest in identifying an antigen, lays the foundation for the diagnosis of HP; however, in more than 50% of patients, the inciting antigen cannot be identified [7]. Pulmonary function tests (PFTs) exhibit restrictive lung pathology while radiological findings range from bilateral interstitial and reticulonodular opacities to a ground glass appearance, and may even appear normal. A typical high-resolution CT (HRCT) shows ground glass opacities along with features of airway involvement (centrilobular nodules or mosaic attenuation) [8]. Bronchoalveolar lavage (BAL) with a lymphocyte count above 50% of the baseline, a neutrophil count above three percent, and a mast cell count above one percent are suggestive of HP [8]. More recent guidelines suggest a cutoff of 30% for lymphocytes which suggests or rules out the diagnosis of HP, while there is no neutrophil or basophil percentage [9]. In the absence of defined clinical guidelines, an integrative approach is often used to diagnose HP in a suspected patient. The mainstay of treating HP is avoidance of antigens while corticosteroids might be required [7].

We present a case of a 46-year-old female with HP, who presented with fever and shortness of breath. As she presented in the pandemic, her condition was initially diagnosed as COVID-19 as a result of anchoring bias due to similar symptoms and radiologic features. A detailed further probing into history revealed the diagnosis of HP and hence, was managed accordingly.

## Case presentation

**Figure 1:**
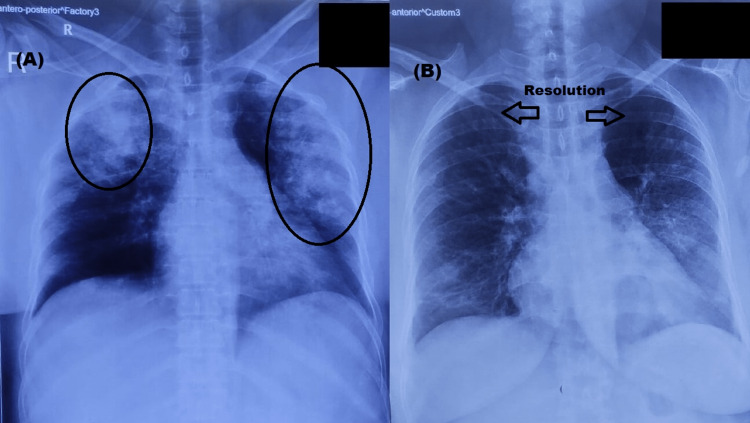
Chest X-ray images of the patient (A): On Presentation: Ill-defined patchy as well as confluent radio-opacities are seen in the subpleural and peripheral locations in bilateral lung fields (left more than right) with perihilar sparing (encircled) (B): Resolution of pathology with treatment

A 46-year-old female presented with fever and shortness of breath for two days. Fever was 38 to 39 degrees Celsius with no diurnal variation. Shortness of breath was acute in onset with no associated complaints of chest pain, palpitations, orthopnea, or abdominal pain. On examination, she had tachycardia, tachypnea, and hypoxemia with an oxygen saturation of 80% measured by a pulse oximeter on room air. Physical examination revealed diffuse scattered wheezing and crackles on chest auscultation and was otherwise unremarkable. Laboratory examination (complete blood count, serum electrolytes, and renal and liver function tests) was within normal range except for an increased leukocyte count of 12,300/microliter. Lactate levels were done in several arterial blood gas (ABG) monitoring and they were not elevated. Procalcitonin levels could not be done in our hospital. Chest X-ray revealed ill-defined patchy as well as confluent radio opacities in subpleural and peripheral locations as shown in Figure 1A. The patient was managed in the medical ICU with antibiotics (ceftriaxone and azithromycin) and non-invasive ventilation (NIV). Reverse transcription-polymerase chain reaction (RT-PCR) for COVID was negative. Nevertheless, in the COVID era, she was managed with the escalation of antibiotics and steroids. The patient’s condition improved within a week and an X-ray of the chest also showed improvement as shown in Figure 1B.

**Figure 2:**
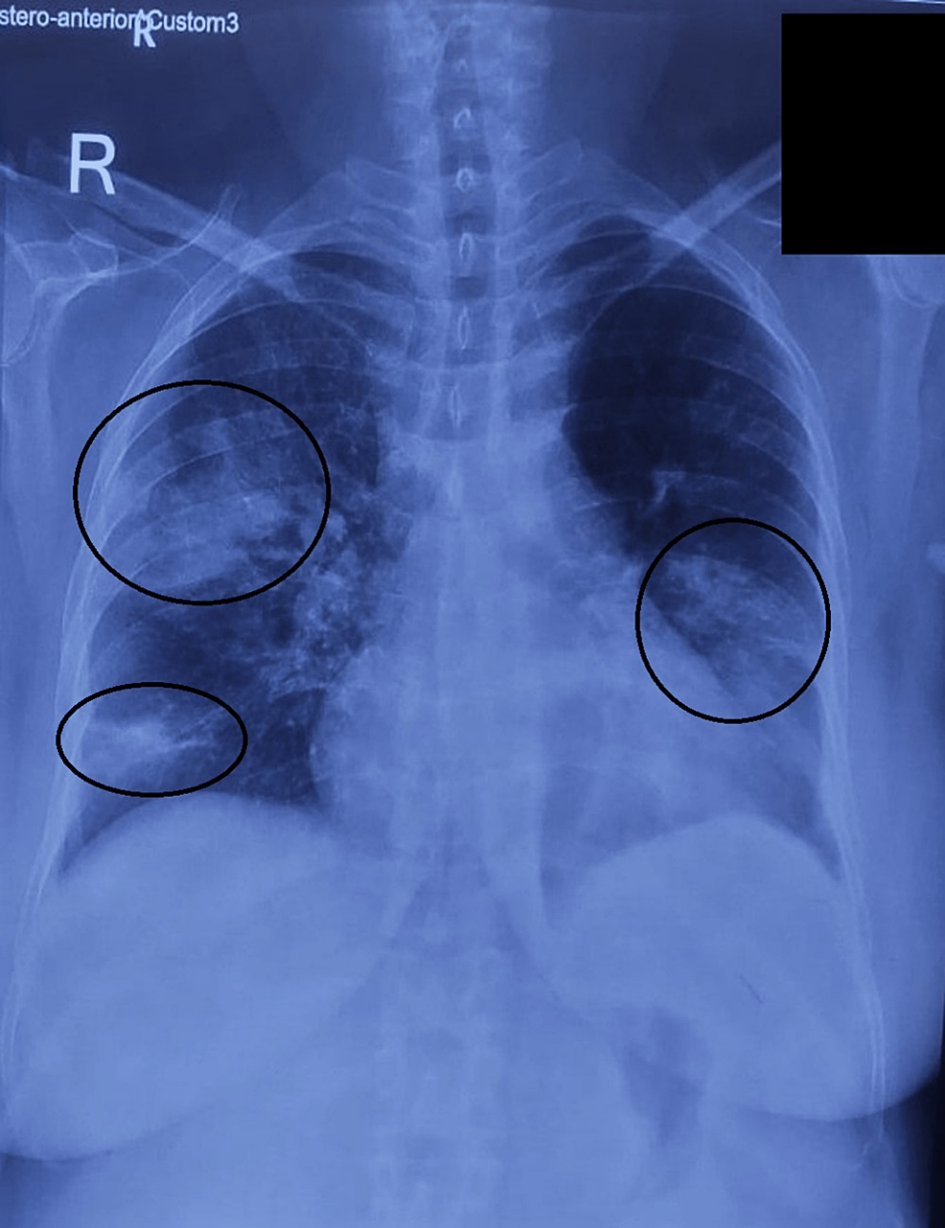
Chest X-ray image of the patient on next presentation Increased reticulonodular shadows in bilateral lung fields (encircled) predominantly in the peripheral and subpleural locations

She was discharged only to present to the hospital within a week later with similar complaints. The X-ray of the chest showed patchy opacities at different locations (Figure 2).

**Figure 3:**
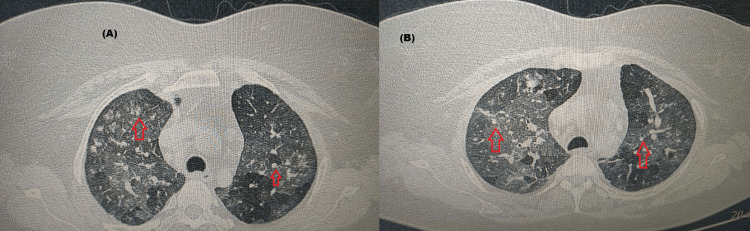
High-resolution CT (HRCT) chest images of the patient at two different CT cuts Figure (A): Multiple centrilobular nodular opacities (red arrow) coalescing to form patches of consolidation Figure (B): Multiple centrilobular nodular opacities (red arrow) Both (A) and (B): mediastinal and hilar lymphadenopathy and multifocal discrete patches of ground glass opacities with intervening areas of normal lung parenchyma giving mosaic attenuation appearance

On further probing into the history and home conditions, it was found that she had several pets including cats and dogs at home. Recently, a cat was acquired as a new pet. HRCT scan of the chest was done which showed multifocal discrete patches of ground glass opacities and multiple centrilobular nodular opacities (Figure 3).

**Figure 4:**
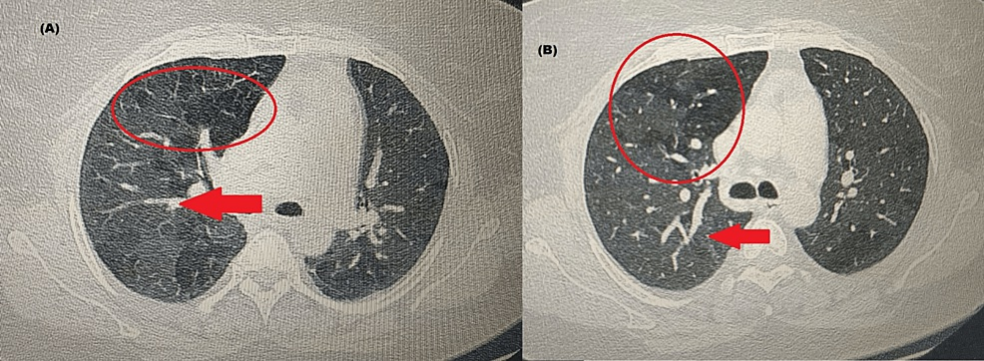
High-resolution CT (HRCT) chest after three months follow-up at two different CT cuts Both (A) and (B) show resolving mosaic attenuation (encircled) in bilateral lung fields and tractional bronchiectasis (arrow) with adjacent fibrocystic and fibroconsolidatory changes

The patient was given methylprednisolone for three days and her condition improved. She was then discharged on a tapering dose of steroids and advised to avoid exposure to cats. After three months, a repeat HRCT was done which showed a significant reduction in ground glass opacities, and centrilobular nodules as compared to the early scan (Figure 4).

## Discussion

HP is a respiratory disorder involving alveolar interstitium, terminal respiratory bronchioles, and alveoli caused by an allergic reaction [7]. Originally classified as acute, subacute, and chronic HP, many other classifications have been proposed which are more clinically relevant. One such classification divides it into acute and chronic HP based on treatment guidelines [3]. Whereas, based on prognosis, another study categorizes HP into fibrotic and non-fibrotic based on radiological imaging [6].

The pathogenesis of HP involves immune complex-mediated (type III) or T lymphocyte-mediated hypersensitivity reaction to inhaled antigens [10]. A large number of antigens have been reported to incite inflammation or fibrosis in HP. These antigens may be non-protein chemicals like isocyanates or may also be polysaccharides, but proteins derived from microorganisms, fungi, plants or animals are the most common inciting antigens reported [5,11,12]. These connect to human proteins to form antigens called haptens [4]. Exposure to these antigens induces both humoral and T helper 1 mediated cell-mediated immune response in sensitized patients [13,14]. It has also been hypothesized that the immune response in normal individuals is not large enough to cause any pathology while in certain genetically susceptible individuals, there is decreased activity of suppressor T cells which leads to an increased and uninhibited immune reaction [15].

Acute HP presents with influenza-like symptoms with complaints of fever, cough, dyspnea, and malaise. It correlates to heavy exposure while subacute and chronic HP relates to prolonged exposure to a previously sensitized antigen. A thorough history must be obtained regarding occupation, hobbies, residence, and pets to identify the antigen, although, in a majority of cases diagnosed with HP, the offending agent couldn’t be found. On examination, tachycardia and tachypnea may be evident. HP presents with usually restrictive but may be an obstructive or mixed pattern with bibasilar crackles with severe cases presenting as hypoxemia and respiratory failure [16]. 

Chest radiographic and CT scan findings vary and depend on the stage of HP. It's better to differentiate fibrotic and non-fibrotic HP radiologically [6]. In acute HP, the predominant finding is ground glass opacity, sometimes with nodules or reticulonodular opacities; whereas, in subacute form, centrilobular nodules and air trapping are seen. Chronic HP presents with honeycombing and reticular opacities [2].

A study showed that positive precipitating antibodies to the offending antigen are a significant predictor of HP with an odds ratio of 5.3 [1]. However, it won’t be prudent to rule in or out HP solely on the basis of the presence or absence of precipitating antibodies. Positive BAL findings of lymphocytosis in ILD patient is suggestive of HP [17]. In the dearth of agreed-upon protocols for diagnosis, a multidisciplinary approach is used to diagnose HP. We made the diagnosis on the basis of clinical history, radiological findings, and recurrence of symptoms after exposure due to cat hair as antigen in accordance with HP.

The primary management in HP is the identification of antigen exposure and avoidance of offending antigen completely (preferred). In severe cases when there is no remission with avoidance of antigen, corticosteroids are used. In cases of chronic HP, once fibrosis appears, it becomes irreversible and lung transplantation may be considered in severe cases.

Due to the devastating incidence and prevalence of COVID-19, the pandemic had a substantial effect on the functioning of the healthcare system. Accelerated morbidity and mortality caused by COVID-19 prioritized ruling it out as a diagnosis, which also led to diagnostic anchoring bias in many cases, thus affecting heuristic decision-making [18]. Cognitive bias is often described as forming and maintaining a presumed diagnosis based on an initial impression, overlooking some parts of history as well as examination. It is a common phenomenon observed among clinicians and even has been reported as bias resulting in misdiagnosis due to the COVID pandemic [19]. Other differentials which could be considered are viral pneumonitis, organic dust toxic syndrome, hot tub lung, chronic bronchiolitis with fibrosis, acute eosinophilic lung disease, and idiopathic pulmonary fibrosis. Many of the abovementioned differentials can be diagnosed based mainly on history, results of sputum and BAL investigations, and HRCT findings. Further investigations such as lung biopsy can be done for confirmation or diagnosis of the disease. This bias can be resolved by focusing more on patient history and examination rather than on investigation-based patient assessment [19].

## Conclusions

In this case report, we emphasize the importance of realizing the cognitive bias, especially in this COVID era as well as detailed history taking. It should also be noted that clinicians should maintain a high index of suspicion towards other pulmonary pathologies even in patients with presentations similar to COVID-19.

We corrected our bias and formed the right diagnosis by elucidating a more detailed history of the patient which led to the correct diagnosis and remission of the patient. Thus, history taking and examination are of paramount importance in any clinical scenario and diagnosis should be framed based on clinical assessment, whereas investigations should mostly be used for confirmation.
